# Evaluation of Microwave Heating Uniformity for Ready-to-Eat Rice in Metalized Packaging Structure

**DOI:** 10.3390/foods13233979

**Published:** 2024-12-09

**Authors:** Chai Liu, Bo Tian, Huiran Liu, Liuyang Shen, Yong Zhu, Chenghai Liu, Xianzhe Zheng, Xiting Deng, Yuxin Zhao

**Affiliations:** 1 College of Engineering, Northeast Agricultural University, Harbin 150030, China; liuchai@neau.edu.cn (C.L.); feiyanghero@163.com (L.S.); liuchenghai@neau.edu.cn (C.L.);; 2 College of Food Science, Northeast Agricultural University, Harbin 150030, China

**Keywords:** microwave reheating, ready-to-eat rice, temperature uniformity, metalized packaging, simulation model

## Abstract

Microwave energy utilization undergoes two stages via absorption and conversion inside ready-to-eat rice (RER) under microwave reheating. The reasonable utilization of microwave energy inside the processed material may enhance the uniformity of the temperature distribution. To analyze the uniformity changes inside RER, the effects of microwave reflection, refraction, and absorption by a metal aluminum film were studied through the thermodynamic properties. A simulation model was developed using the co-simulation method of COMSOL Multiphysics with MATLAB programming to analyze the mechanism of material properties and electromagnetic distribution on temperature distribution uniformity, as well as the formation mechanism of the temperature distribution uniformity of microwave-reheated RER. Based on models of the designed package boxes covering the metal film, the optimal structure was developed to include a metal aluminum film with a width of 5 mm and a thickness of 0.30 mm, which was sprayed on the edges and corners of a rectangular packaging box. The packaging boxes covering the metal films may reduce the average temperature of the upper and lower layers in RER by 8.03 °C and 7.42 °C, respectively, while the temperature distribution uniformity increased by 35.71% and 72.22%. The introduction of a metalized package significantly enhances the temperature uniformity inside RER under microwave reheating.

## 1. Introduction

A variety of ready-to-eat meals (from frozen or refrigerated dishes [1]) may offer delicious, convenient, and quick options for consumers [2]. Microwave reheating is a crucial operation in the processing of ready-to-eat meals, which has advantages over the traditional reheating methods in efficiency, convenience, and hygiene [3]. However, the unclear mechanism of microwave reheating non-uniformity, combined with the ready-to-eat meals’ unstable quality change, result in poor palatability after reheating, which is the primary technological bottleneck restricting its processing and application. The uniformity of the temperature distribution inside ready-to-eat meals under microwave reheating was determined by the shape of the heated food, material characteristics, and reheating process parameters [4]. When spherical-shaped food was heated, the microwave energy was easily concentrated in the center (central focus effect), resulting in an excessively high temperature [5]. The center in the circular cross-section and the quadrilateral’s longitudinal section of the cylindrical food appeared as hot spots under reheating [6]. For rectangular-shaped food, under reheating, the microwave energy was focused around the edges and corners (wall and corner effect), causing a rapid elevation in temperature [7]. An appropriate design of food size paired with the microwave penetration depth helped to improve microwave reheating effects. The analysis of the physical properties of food enables us to understand the heat and mass transfer mechanism of food during the microwave reheating process [8]. The thermal properties of food represented its ability to convert microwave energy into thermal energy and transport heat from an interior high-temperature zone to a low-temperature region. The specific heat capacity and thermal conductivity were key considerations in the refrigeration, drying, and heating processes [9]. The dielectric properties of food determined its ability to absorb and convert microwave energy. The higher dielectric properties of food resulted in the more effective absorption of microwave energy with a high reheating speed [4]. The optimization of microwave reheating process parameters, such as the reheating time and microwave power, can ensure the quality and technical parameters of ready-to-eat meals [10].

To analyze the generating mechanism of uniform microwave reheating and the quality stability of ready-to-eat meals, both domestic and international scholars have examined the topic from the perspectives of material characteristics (composition, dielectric properties, thermal properties, geometric shape, and size), microwave cavity structure (structure and size of microwave cavity, position and arrangement of food), packaging form (microwave inert packaging, microwave active packaging, microwave sensor packaging, packaging pattern, packaging structure, packaging size, etc.), material movement mode (static, translational and rotational motion, etc.), and microwave reheating process (microwave power, reheating time, intermittent ratio, etc.) [11,12,13]. In the current case, the advancement of microwave reheating uniformity is mainly focused on the following three areas.

(1) Dietary combinations shift from single to multi-component foods.

Microwave reheating materials have developed from simple geometric forms and single food simulants (agar) [14,15] to diverse shapes and multi-component food (for example, mashed potatoes, rice, chicken and seasonings, etc.) in a variety of ways (for example, up and down, left and right arrangement, interval placement, etc.) [16,17,18]. It has been revealed that, due to the complexity of microwave reheating properties, food composition, and food shape, the food in the microwave reheating process displays noticeable non-uniform heating phenomena, such as the ‘central focus effect’ (the circular center obtains the highest temperature) and ‘wall and corner effects’ (the rectangular corners obtain the highest temperature) [5,19]. Due to their numerous components and obvious differences in physical properties (density, moisture content, thermal properties, dielectric properties, etc. [4]), foods that are fully mixed or that combine multiple ingredients have complex heat and mass transfer mechanisms under microwave reheating [20,21], which is a hot and difficult subject of current research.

(2) Microwave reheating properties are analyzed to develop a control mechanism model.

Existing research has employed Multiphysics coupling simulation software (COMSOL Multiphysics 5.5) to analyze the electromagnetic field distribution, microwave absorption, and heat and mass transfer processes in the microwave food reheating process based on the simulation analysis method [22,23]. Nevertheless, the simulation and bench test fit poorly, and few investigations have been conducted into the material’s translational or rotational motion within the microwave cavity [24,25,26]. As a result, a mathematical model of the microwave reheating process and packaging material parameters were developed, as well as an investigation into the process of reheating ready-to-eat meals (rotational motion). To avoid the impact of uneven temperature distribution on the taste and quality of microwavable food, the temperature distribution under microwave reheating was adjusted based on the microwave reheating parameters and packaging material characteristics.

(3) From parameter optimization to quality control.

The metalized packaging design could reduce the non-uniformity of the temperature distribution under microwave-reheated ready-to-eat meals by utilizing the reflection and refraction of microwave-active materials in microwaves [27]. The majority of research has focused on the design of metalized packaging structures for single-component food to weaken the central focus effect (for circular food, the microwave energy is focused in the center during reheating, resulting in a higher temperature in the circular center than in the other regions [5]) or wall and corner effects (for rectangular food, the microwave energy gathered around the edge and corner of the rectangular food during reheating, resulting in a higher temperature around the edges and corners than in the other regions, with corners having the highest temperature [7]), but there were no corresponding quality control theories or optimization methodologies [28,29]. The foil-coated packaging form of the reheating container was designed to control the transmission and distribution of electromagnetic waves during microwave reheating, using the features of microwave-inert and active materials [30]. The analysis of the heat and mass transmission mechanisms inside food under microwave reheating helps with the optimization of the reheating container’s parameters and metalized film packaging form. This improved the efficiency of microwave energy utilization and the uniformity of the food’s temperature distribution. Previous research has offered a theoretical foundation for enhancing microwave reheating uniformity and regulating the quality of reheating meals composed of several components.

Based on the above-mentioned research, metalized packaging structures were developed to enhance the uniformity of microwave reheating for ready-to-eat meals. The packaging box made of microwave-active materials could alter the electric field distribution in ready-to-eat meals during microwave reheating [11,31]. A reasonable structure design of an active packaging box could avoid the situation in which the edge or center of the ready-to-eat meals were not heated during reheating [4,32]. It might also lessen the difference in temperature between the ‘hot spots’ and the ‘cold spots’ of heated ready-to-eat meals, aid in meeting food safety minimum temperatures, and increase the palatability of ready-to-eat meals [29]. Three effects of microwave-active materials on microwaves were shielding, field adjustment, and sensory absorption [11]. The shape and size of a tray made of microwave-active materials affected the uniformity of the temperature distribution during microwave reheating [33]. To achieve a uniform temperature distribution, the bottom and sides of the rectangular packaging box were covered with shielding elements (made of microwave shielding material) and diffusion elements (made of microwave transmission or reflective material) to obtain a uniform temperature distribution [34]. It has been discovered that the width, spacing, thickness, and angle between the microwave-active material and the bottom surface affect the change in the electric field distribution [35,36]. RER is the staple food in ready-to-eat meals [37]. The RER was packaged in a rectangular polypropylene (PP) packaging box to increase the loading space while improving storage stability [11]. It was suitable for transportation, storage, and sales under cool chain conditions, and could be eaten after reheating [38]. Conventional reheating methods included cooking, microwaving, heating packages, and so on [39]. The reasonably metalized packaging box could effectively alleviate the non-uniform temperature distribution inside the RER [4]. The research objectives of this study are as follows:

(1) To build a microwave simulation model for the microwave reheating of refrigerated RER in a household microwave oven.

(2) To compare the four microwave simulation models that used metal aluminum film to increase the RER’s temperature under microwave reheating.

(3) To investigate the reasons for the microwaves’ ‘wall and corner effects’ within the rectangular-shaped RER and improve its reheating uniformity.

## 2. Materials and Methods

To clarify the influence mechanism of temperature distribution uniformity in the microwave reheating process of RER, and to provide a reasonable basis for the uniformity enhancement of the temperature distribution, a microwave reheating bench test and a numerical simulation of RER were carried out in a microwave oven, based on the already measured and analyzed thermal physical parameters of RER [4]. Establishing a three-dimensional simulation model of RER under microwave reheating, it was possible to view the RER’s temperature after reheating, and quickly and intuitively understand the microwave energy absorption and temperature distribution of the RER [5].

Analyzing the temperature distribution of RER after microwave reheating could provide a basis for enhancing the temperature distribution uniformity. The main steps in the numerical simulation of the microwave reheating process of RER using COMSOL 5.5 Multiphysics software include building the geometric model, setting the physical field and input parameters, applying the simulation method, and supplying the boundary conditions [40]. In the analysis, the spatial electric field and temperature distribution of microwave-reheated RER could be obtained by inputting the material characteristic parameters, selecting the appropriate physical field module, setting the grid, and calculating the solution method [40]. The simulation results were utilized to analyze the temperature change rule and temperature distribution non-uniformity of RER.

However, it was difficult to simulate the motion process of RER due to the rotation of the glass turntable within the microwave oven, which rotated during the microwave reheating process [41]. The RER rotated with the glass turntable in the microwave field, and the dynamic solution of the physical field was one of the technical difficulties in the numerical simulation of moving materials during the microwave reheating process. Furthermore, previous research discovered that the optimal process parameters for the microwave reheating of RER were as follows: microwave power of 800 W and reheating time of 180 s [4]. As a result, the microwave reheating bench test of RER was carried out by employing the process parameters, and the simulation model was verified. The temperature distribution and uniformity change rule of RER in the microwave reheating process were analyzed using the bench test and simulation results, as well as the simulation method and the proposed simulation strategy for optimizing the microwave reheating process of the RER in the motion state.

### 2.1. Microwave Reheating Model Establishment

#### 2.1.1. Geometric Model

**Figure 1:**
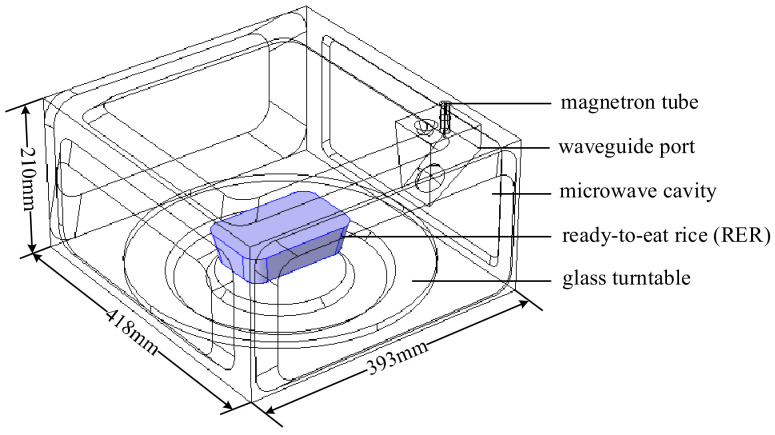
The microwave reheating geometric model schematic diagram for RER. The PP rectangular packaging box had a bottom size of 138 × 86 mm^2^ and a height of 44 mm.

A microwave reheating RER model was built in COMSOL 5.5 software utilizing the primary structural parameters of the experimental microwave workstation (Panasonic NN-ST780 W microwave oven, Kadoma, Japan), as illustrated in Figure 1. The microwave reheating model was made up of a microwave oven cavity, a waveguide, a glass turntable, the RER, and an RER packaging box. The RER and its packaging box were placed in the center of the glass turntable, with the long side perpendicular to the waveguide. The glass turntable rotated at a speed of 12 r/min throughout the microwave cooking process. The microwave power was set to 800 W and the reheating time was set to 180 s. The waveguide for reheating RER directed microwave energy into the workstation, and the microwave focusing effect was investigated to propose a strategy for reducing the ‘wall and corner effects’ with a metal aluminum film.

#### 2.1.2. Model Assumptions

The following assumptions were considered to improve the computational efficiency and simplify the simulation process for the RER model under microwave reheating.

(1) In the model, the microwave oven cavity and waveguide were composed of copper, but their thickness and heat loss were neglected.

(2) The waveguide’s transverse electric wave propagated without an electric field.

(3) The RER was an isotropic material; the initial temperature and moisture were uniform.

(4) The air and glass turntable in the microwave workstation did not conduct heat.

(5) Water transfer was not considered, the microwave reheating process was brief, and the RER’s temperature was less than 100 °C during the whole process [4].

(6) The RER was not distorted throughout the microwave reheating process.

#### 2.1.3. Governing Equations

(1) Electromagnetic field control equations

The temperature change in RER under the microwave reheating process altered the material characteristic index of each component, which in turn affected the temperature distribution. The interaction between the microwave and RER was determined by a combination of electromagnetic and heat forces. As illustrated in Equations (1) and (2), microwave transmission followed the Maxwell electromagnetic field equation, which theoretically characterized the variation in electromagnetic waves with time and space.

(1) ∇×E=−jωμH

(2) $$\nabla \times H=j\omega \varepsilon_{0}\varepsilon E$$

where E is the electric field intensity (V/m), ω is the angle frequency (rad/s), μ is the electromagnetic flux (Wb), *H* is the magnetic field intensity (A/m), $\varepsilon_{0}$ is the vacuum dielectric constant, with $\varepsilon_{0}=8.854\times 10^{-12}$ F/m; ε is the relative dielectric constant, the real part ($\varepsilon^{\prime }$) characterizes RER’s ability to store energy throughout the polarization process, and the imaginary part ($\varepsilon^{\prime \prime }$) characterizes the RER’s energy loss during the polarization process.

The electromagnetic field was regulated by the frequency domain equation and calculated using Equation (3).

(3) $$\nabla \times \mu_{r}^{-1}\left(\nabla \times \vec{E}\right)-k_{0}^{2}\left(\varepsilon_{r}-\frac{j\sigma }{\omega \epsilon_{0}}\right)\vec{E}=0$$

where $k_{0}$ is the wavenumber of free space ($k_{0}=2\pi /\lambda_{0}$) and σ is the electric conductivity (S/m).

Both the electric and magnetic field strengths were defined as functions of time in Equations (4) and (5).

(4) $$\vec{E}\left(x,y,z,t\right)={\vec{E}}_{0}\left(x,y,z\right)e^{j\omega t}$$

(5) $$\vec{H}\left(x,y,z,t\right)={\vec{H}}_{0}\left(x,y,z\right)e^{j\omega t}$$

(2) Microwave energy absorption

When exposed to microwave radiation, the polar molecules within the RER relaxed in orientation, resulting in friction and collision. The microwave energy was transformed into heat energy, raising the temperature of the material. The Poynting theory was used to compute the RER’s absorption of microwave energy throughout the microwave reheating process, and Equation (6) was used to determine the electromagnetic loss per unit volume of RER.

(6) $$Q=2\pi f\varepsilon_{0}\varepsilon^{\prime \prime }{\left|\vec{E}\right|}^{2}$$

where *Q* is the microwave energy absorbed by RER in W/m^3^ and f is the microwave frequency, with a value of 2.45 GHz.

(3) Microwave energy utilization

During the microwave reheating process, the heat volume generated by the microwave energy inside the RER equals the sum of the heat accumulated inside the RER, the heat transmitted, and the heat consumed during the water transfer and evaporation processes. The RER’s heat transmission differential equation can be calculated using the law of energy conservation and the Fourier law, as illustrated in Equation (7).

(7) $$\rho C_{P}\frac{\partial T}{\partial t}+\nabla \cdot \left(-K\nabla T\right)=Q-\rho C_{P}\nu \cdot \nabla T$$

where ρ is the density of the RER in kg/m^3^, $C_{p}$ is the RER’s specific heat capacity in J/(kg·K), T is the temperature of the RER in °C, K is the thermal conductivity of the RER in W/(m·K), and ν is the velocity vector in m/s.

The heat loss was represented by the surface convective heat transfer coefficient. Equation (8) depicts the boundary conditions for convective heat transfer between the RER surface and the surrounding air in the microwave workstation.

(8) $$-k\nabla T=h_{c}\left(T-T_{a}\right)$$

where $h_{c}$ is the surface convection thermal transfer coefficient in W/(m^2^·K) and $T_{a}$ is the temperature of the air in a microwave workstation (°C).

(4) Sufficient and necessary conditions

The microwave workstation and waveguide are made of copper. The electric field strength is zero, and the boundary condition is shown in Equation (9).

(9) n×E=0

The initial temperature was uniform while calculating the RER’s transient temperature field during the microwave reheating process, as indicated in Equation (10).

*t* = 0, *T* = *T*_0_ = 4 °C (10)

When a metal material is placed in an electromagnetic field, the Maxwell equations in the frequency domain form (Equation (11)) must be considered.

(11) $$\nabla \times \left(\mu_{r}^{-1}\nabla \times E\right)-\frac{\omega^{2}}{c_{0}^{2}}\left(\varepsilon_{r}-\frac{i\sigma }{\omega \varepsilon_{0}}\right)E=0$$

where $\mu_{r}$ is the relative permeability in H/m.

The addition of a lossy material (aluminum film) required the introduction of a virtual numerical term in the control equation of the COMSOL 5.5 software. Under high frequencies, the aluminum film material approached plasma resonance [32]; the current in the lossy material would be generated and pushed to the border by electromagnetic induction; and the skin depth can serve as a depth measuring index of the current entering the material [42]. Equation (12) was used to calculate the skin depth. This was the distance into the material when the current was reduced to 1/e [43].

(12) $$\delta =\frac{1}{\sqrt{\pi f\sigma \mu_{0}\mu_{r}}}$$

where $\mu_{0}$ is the magnetic permeability of the vacuum, with a value of 1.257 × 10^−6^ H/m.

The aluminum film’s skin depth must be determined when establishing the boundary conditions, and the feature size (*L*c) of the skin depth value simulation object was contrasted [42]. *L*c could be defined as the volume-to-surface area ratio or the thickness of the object’s thinnest component; in this study, *L*c was defined as the object’s thinnest part. Currents would enter the object, the skin effect would push these currents to the surface, and the impedance boundary condition would be employed when *L*c was substantially more than δ. The impedance was defined by the film thickness and the tangential impedance, whereas the transition boundary conditions employ the material properties and film thickness as inputs. The theoretically stated relationship between the induced surface current density and the electric field discontinuity assumed that the electromagnetic wave propagated along the normal direction in the thin layer (Equations (13)–(17)) [44].

(13) $$J_{s1}=\frac{\left(Z_{S}E_{t1}-Z_{T}E_{t2}\right)}{{Z_{S}}^{2}-{Z_{T}}^{2}}$$

(14) $$J_{s2}=\frac{\left(Z_{S}E_{t2}-Z_{T}E_{t1}\right)}{{Z_{S}}^{2}-{Z_{T}}^{2}}$$

(15) $$Z_{S}=\frac{-j\omega \mu }{k}\frac{1}{\tan\left(kd\right)}$$

(16) $$Z_{T}=\frac{-j\omega \mu }{k}\frac{1}{\tan\left(kd\right)}$$

(17) $$k=\omega \sqrt{\left(\varepsilon +\left(\frac{\sigma }{j\omega }\right)\right)\mu }$$

where $J_{s}$ is the current density (surface) in A/m, $Z_{S}$ is the surface impedance in Ω, $Z_{T}$ is the transfer impedance in Ω, $E_{t}$ is the tangential electric field (surface) in V/m, and k is the wave number.

#### 2.1.4. Input Parameter Setting

In the model, the waveguide port was set to TE10, the microwave frequency was 2.45 GHz, and the input power was 800 W. The initial temperature of the air and glass turntable in the microwave oven was 20 °C, while the initial temperature of the RER was 4 °C [4]. Table 1 shows the properties of the air, glass turntable, copper, and aluminum, as retrieved from the COMSOL material library. The material properties of RER have been studied in previous research, as shown in Table 2 [4].

#### 2.1.5. Meshing Scheme

The mesh division determined the rationality of numerical simulation results: the smaller the mesh size, the more precise the solution and the greater the convergence [45]. However, as the size of the mesh unit decreased, the time and computer storage requirements for the solution dramatically increased [20]. As a result, establishing an acceptable mesh size will improve the consistency of model solution results, shorten model solution time, and reduce computer storage requirements. The COMSOL Multiphysics 5.5 software could automatically adjust the mesh size for each medium in the model based on the wavelength shift in the different media. Because the dielectric characteristics of the medium change with temperature, the mesh size must be customized frequently. The mesh size was defined as shown in Equation (18).

(18) $$1\leq m_{mesh}\leq \frac{c}{Mf\sqrt{\varepsilon_{max}^{’}}}$$

where $m_{\mathrm{mesh}}$ is the mesh size of the medium in the model (mm), c is the microwave speed (vacuum), f is the microwave frequency, with a value of 2.45 GHz, $\varepsilon_{max}^{\prime }$ is the real part’s maximum value of the dielectric constant, and *M* is the mesh number in each microwave wavelength.

The normalized power absorption (*NPA*) may be used to estimate the grid size. The electromagnetic power loss density in the material varied with the mesh size. If it did not change considerably as the mesh size increased, this implied that the mesh size could be used to produce more accurate results [46]. Equation (19) depicts the *NPA* definition of the microwave.

(19) $$NPA=\frac{P_{ab}}{P_{in}}$$

where $P_{ab}$ is the absorption power of the material (W) and $P_{in}$ is the input power of the model (W).

The microwave oven cavity, coaxial cable, material, and glass all have different dielectric constants. The solution time was set to 1 s, and the grid number in a single microwave wavelength for different materials was steadily increased from 0.5 to 11, at which point the computation was completed. The data values for each medium’s maximum unit size, total mesh number, computing time, simulated material absorption power, and input power are shown in Table 3.

**Figure 2:**
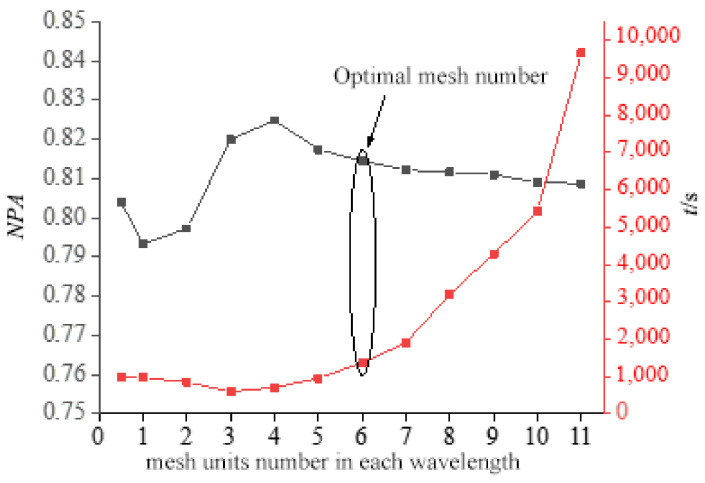
The influence of the mesh unit number in each wavelength on *NPA* and solution time. The optimal mesh number, where the *NPA* tended to be stable with reduced solution time, was indicated by the circle in the figure.

**Figure 3:**
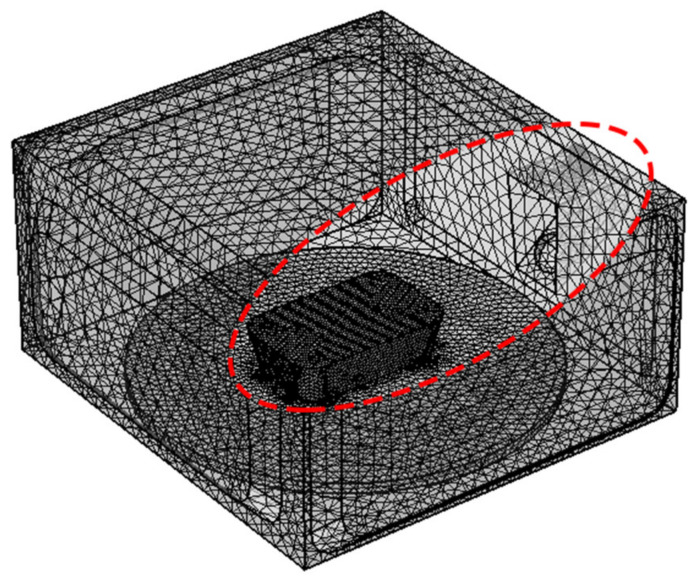
Schematic diagram of the overall mesh division in microwave reheating systems. (The red circle was the optimal mesh number.).

As shown in Figure 2, the *NPA* and solution time changed as the mesh unit number increased. The *NPA* tended to be stable after dividing each microwave wavelength of the material into six mesh units, and the solution time was less than that of a more exact division. As a result, each microwave wavelength was divided into six mesh units, with the mesh size determined by the material. Figure 3 depicts the entire mesh distribution map after dividing each microwave wavelength into six grid units.

#### 2.1.6. Simulation Method

**Figure 4:**
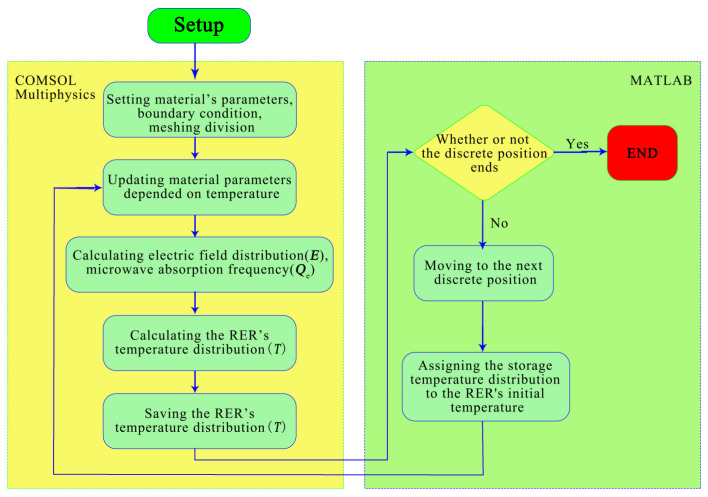
Flowchart for rotational simulation of RER microwave reheating.

**Figure 5:**
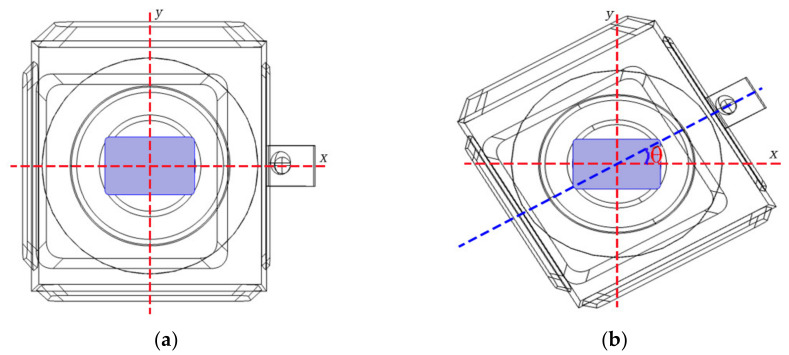
Relative position diagram of the RER and microwave oven cavity rotation. (**a**) During the initial rotation period, the rotation angle of the first discrete position was 0 degrees. (**b**) During the initial rotation period, the rotation angle of the second discrete position was θ. The RER simulation model’s rotation angle between two adjacent discrete positions was θ (θ = 30°).

The RER was placed in the center glass turntable of the microwave oven and rotated at a constant speed until the reheating process was completed. The co-simulation method of COMSOL Multiphysics 5.5 and MATLAB R2022b software replicated the actual reheating operation in the microwave oven. The rotating process was discretized, so the RER was rotated to a specific angle (θ) and heated at a single location. The whole rotation period was divided into *N* positions for reheating, and Figure 4 shows the rotating simulation flow chart. The COMSOL 5.5 software calculated the electric field distribution and absorption power, which were then mapped to the heat transfer module to calculate the temperature distribution *T* based on the supplied material parameters, according to the input material parameters. MATLAB R2022b software was used to decide whether the simulation phase was complete; then, the microwave oven cavity was rotated to the next discrete position, and the initial temperature was adjusted to match the previous discrete position’s final temperature. COMSOL 5.5 software was used to calculate and update the material parameters. COMSOL 5.5 and MATLAB R2022b software were used to repeat the above steps until the reheating was completed. Figure 5 depicts the rotation of both the RER and the microwave oven cavity.

Equation (20) depicts the relationship between the discrete angle (θ) and the discrete position (*N*) during a period.

(20) $$N=\frac{360{}^{\circ}}{\theta }$$

Each rotational cycle in the reheating process lasted 12 s; hence, the reheating time at each discrete location could be expressed using Equation (21).

(21) $$t=\frac{12}{N}$$

It was proposed that a discrete angle of 30° produced better processing results during the model simulation procedure [47]. As a result, the model’s discrete angle was 30° and the reheating time for each discrete position was 1 s.

### 2.2. Model Verification

#### 2.2.1. Evaluation Index for the Model

(1) RER Preparation

Fresh rice (No. 2 fresh rice, Dr. Shang Inc., Harbin, China) was stored in a 4 °C refrigerator for 24 h before being washed three times, soaked for 30 min at a rice-to-water ratio of 1:1.3 (g/g), and cooked with an electric cooker (SR-CW15, Panasonic Inc., Osaka, Japan) [4,48]. A 650 mL rectangular packaging box was filled with 540 ± 20 g of cooked rice. The boxed rice’s surface was kept smooth, sealed, and cooled to room temperature, and then stored for 24 h at 4 °C [4,6].

(2) Measurement position setting

**Figure 6:**
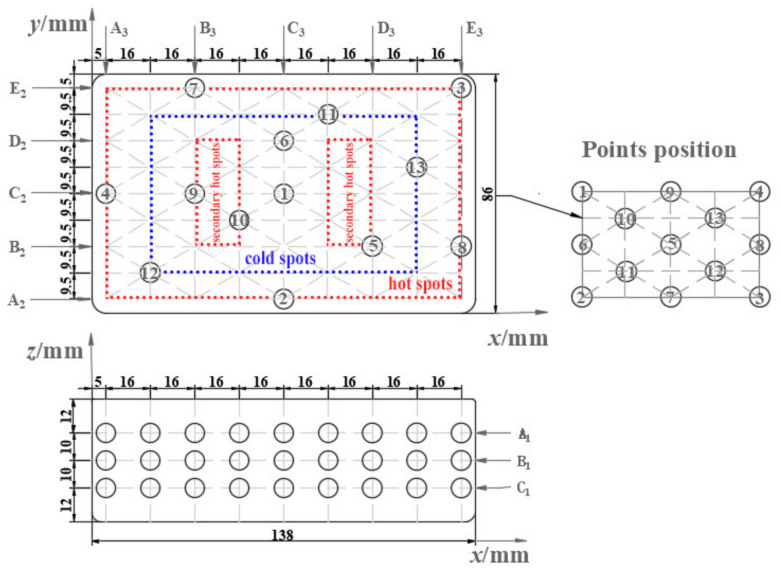
Arrangement scheme for RER measurement points. The packaging box’s layers were all axisymmetric rectangles, and the 13 points were equivalent to 45 measurement points at each layer, representing the entire layer of data.

Figure 6 depicts the RER’s measurement points. The RER was divided into three layers along the *z* direction, named A_1_ (the upper), B_1_ (the middle), and C_1_ (the lower) layers, with 13 measurement points for each layer. The layers were divided into ‘hot spots’, ‘secondary hot spots’, and ‘cold spots’ according to the temperature distribution of the RER’s microwave reheating procedure [4].

(3) Measuring method

The microwave power was set to 800 W, and the reheating time was set to 180 s. Each measurement layer’s temperature for the bench test was measured using an infrared thermal imager (FLIR E95, FLIR Inc., Tigard, OR, USA). The experimental temperature, simulated temperature, and simulated electric field data were measured when the reheating time was 30, 60, 90, 120, 150, and 180 s, respectively.

(4) Evaluation index for electric field distribution

The simulation model was used to visually examine the effect of metalized packaging on the RER’s internal electric field distribution and to find the most effective way to improve the uniformity of the temperature field distribution. During the microwave reheating process, the RER was measured at 30, 60, 90, 120, 150, and 180 s. The probe method was employed to measure the electric field intensity values at each measuring point on the A_1_, B_1_, and C_1_ layers along the *z* direction (*x*-*y* planes). Equation (22) was used to determine the average electric field of each layer (with various *x*-*y* planes), and Equation (23) was used to calculate the holistic average electric field of the RER along the z direction (*x*-*y* planes).

(22) $${\bar{E}}_{1}=\frac{1}{13}\sum_{m=1}^{13}E_{m}$$

(23) $${\bar{E}}_{2}=\frac{1}{3\times 13}\left(\sum_{i=1}^{13}\left(E_{i}\right)+\sum_{j=1}^{13}\left(E_{j}\right)+\sum_{k=1}^{13}\left(E_{k}\right)\right)$$

where ${\bar{E}}_{1}$ is the layer average electric field of the RER (V/m) and ${\bar{E}}_{2}$ is the average electric field of the whole RER (V/m). $E_{m}$, $E_{i}$, $E_{j}$, and $E_{k}$, refer to the electric field at 1 ~ 13 points in each layer (A_1_, B_1_, and C_1_) (V/m).

The uniformity of the electric field distribution in the RER was assessed using the ratio of the standard deviation to the average electric field. Equation (24) was used to obtain the layer electric field uniformity coefficient, which measured the RER’s layer uniformity in the electric field distribution (*x*-*y* planes) [44].

(24) $$COV_{1}=\frac{\sqrt{\frac{1}{13}\sum_{i=1}^{13}{\left(E_{i}-\bar{E}\right)}^{2}}}{\bar{E}}$$

where $E_{i}$ is the electric field at 1~13 points in the layer (V/m) and $\bar{E}$ is the layer average electric field (V/m).

The electric field intensity uniformity of the A_1_, B_1_, and C_1_ layers along the *z* direction (*x*-*y* plane) was utilized to evaluate the electric field uniformity of the entire RER. As shown in Equation (25), the overall electric field uniformity coefficient characterized the uniformity of the RER’s overall electric field distribution.

(25) $$COV_{2}=\frac{\sqrt{\frac{1}{3\times 13}\left(\sum_{i=1}^{13}{\left(E_{i}-\bar{E}\right)}^{2}+\sum_{j=1}^{13}{\left(E_{j}-\bar{E}\right)}^{2}+\sum_{k=1}^{13}{\left(E_{k}-\bar{E}\right)}^{2}\right)}}{\bar{E}}$$

(5) Evaluation index for temperature distribution

The temperature data for the RER were collected at reheating times of 30, 60, 90, 120, 150, and 180 s, and the average temperature and temperature uniformity of the RER’s A_1_, B_1_, and C_1_ layers were calculated along the *z* direction (*x*-*y* plane). Equation (26) was introduced to calculate the layer temperature uniformity coefficient, and Equation (27) was used to calculate the overall temperature uniformity coefficient [49].

(26) $$COV_{3}=\frac{\sqrt{\frac{1}{N}\sum_{i=1}^{N}\left(T_{i}-\bar{T}\right)}}{\bar{T}-T_{0}}$$

(27) $$COV_{4}=\frac{\sqrt{\frac{1}{n\times N}\sum_{i=1}^{n}\sum_{j=1}^{N}\left(T_{ij}-\bar{T}\right)}}{\bar{T}-T_{0}}$$

#### 2.2.2. Model Accuracy Measurement

To evaluate the accuracy of the microwave reheating RER simulation model, the measured RER temperature values were compared to the simulation results, and the root mean square error (*RMSE*) was calculated, as shown in Equation (28).

(28) $$RMSE=\sqrt{\frac{1}{N}\sum_{i=1}^{N}{\left(T_{si}-T_{ei}\right)}^{2}}$$

where *T*_si_ is the temperature at the sampling point via the simulation (°C), *T*_ei_ is the temperature at the sampling point via the experiment (°C), and *N* is the number of sampling points in the reheating process.

#### 2.2.3. Model Verification Results

**Figure 7:**
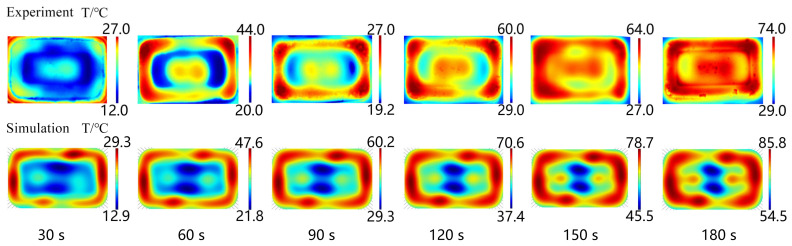
The experimental and simulated temperature distributions of the RER’s layer C_1_. We employed a microwave power of 800 W, a microwave frequency of 2.45 GHz, and the reheating times were 30, 60, 90, 120, 150, and 180 s, respectively. The PP rectangular packaging box had a bottom size of 138 × 86 mm^2^ and a height of 44 mm. The RER’s moisture content was 63.23 ± 0.87%, and its thermal and dielectric properties varied with temperature [4].

**Figure 8:**
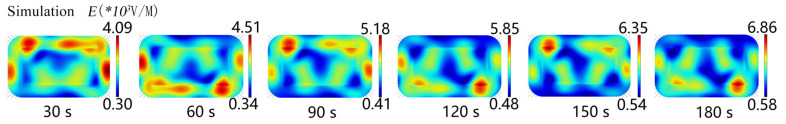
Electric field distribution on the C_1_ layer of an RER simulation model. The PP rectangular packaging box had a bottom size of 138 × 86 mm^2^ and a height of 44 mm. The microwave power was 800 W, the microwave frequency was 2.45 GHz, and the reheating times were 30, 60, 90, 120, 150, and 180 s, respectively.

The accuracy of the microwave reheating RER simulation model was evaluated by comparing the experiment’s temperature to the simulation values, and the experiment was carried out using a microwave workstation (MWS, FLIR Inc., USA) [4]. Figure 7 displays the experimental and simulated temperature distribution of the RER’s layer C_1_ along the *z* direction (*x*-*y* plane) during the microwave reheating operation. Both the experimental and simulated temperatures exhibited obvious wall and corner effects. The incident TE wave paralleled layer C_1_, resulting in non-resonant scattering and electric field concentration in the boundary layer. As the incident TE wave was attenuated in layer C_1_, the electric field distribution in layer C_1_ (Figure 8) generated an alternating change with a half-wavelength interval. Layer C_1_ exhibited ‘cold spots’ in the center, ‘hot spots’ at the corners, and ‘secondary hot spots‘ at a distance equal to the penetration depth inside the RER [50].

**Figure 9:**
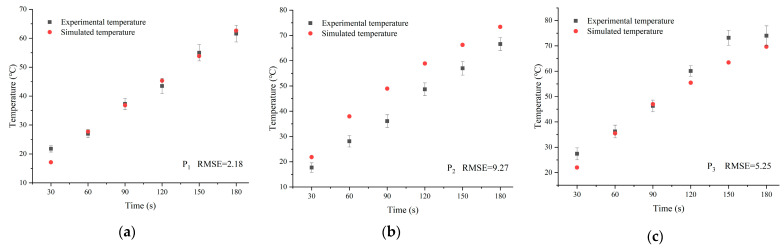

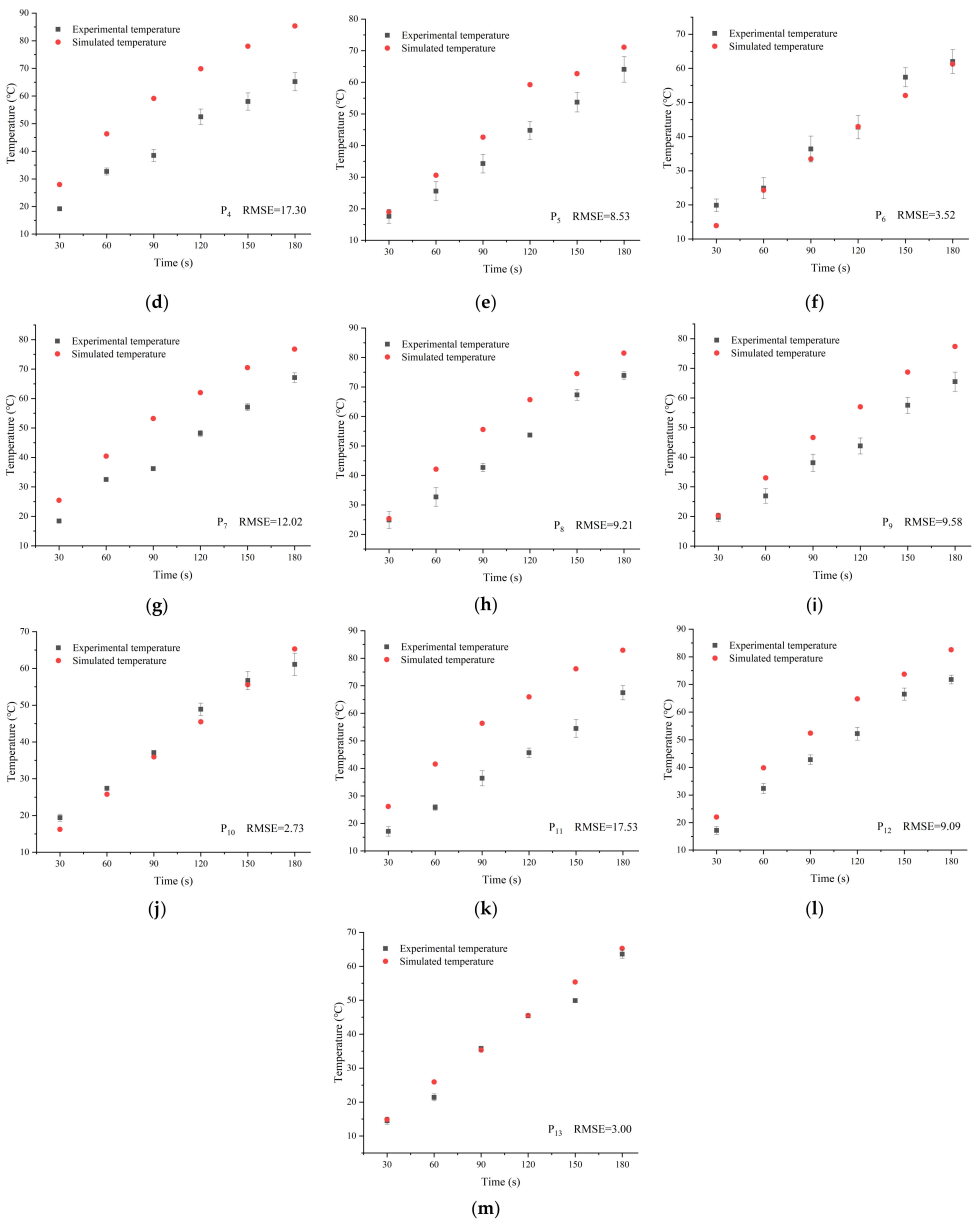
A comparison of simulated and experimental temperatures at 1–13 points in the layer C_1_ of RER. (**a**) Point 1’s comparison data during microwave reheating with an *RMSE* of 2.18; (**b**) point 2’s comparison data during microwave reheating with an *RMSE* of 9.27; (**c**) point 3’s comparison data during microwave reheating with an *RMSE* of 5.25; (**d**) point 4’s comparison data during microwave reheating with an *RMSE* of 17.30; (**e**) point 5’s comparison data during microwave reheating with an *RMSE* of 8.53; (**f**) point 6’s comparison data during microwave reheating with an *RMSE* of 3.52; (**g**) point 7’s comparison data during microwave reheating with an *RMSE* of 12.02; (**h**) point 8’s comparison data during microwave reheating with an *RMSE* of 9.21; (**i**) point 9’s comparison data during microwave reheating with an *RMSE* of 9.58; (**j**) point 10’s comparison data during microwave reheating with an *RMSE* of 2.73; (**k**) point 11’s comparison data during microwave reheating with an *RMSE* of 17.53; (**l**) point 12’s comparison data during microwave reheating with an *RMSE* of 9.09; and (**m**) point 13’s comparison data during microwave reheating with an *RMSE* of 3.00. The *RMSE* value was calculated using Equation (28).

Figure 9 depicts the temperature from 1 to 13 points in layer C_1_ of the experiment and simulation for the RER under microwave reheating. The simulated temperature was usually higher than the experimental temperature due to neglecting the electromagnetic wave lag in the microwave oven, as well as the heat transmission between the RER and the surrounding air and the glass turntable. The temperature distributions in the simulation and experiment were consistent, and the model’s accuracy met the requirements.

## 3. Results and Analysis

Research into metalized packaging for microwave reheating processes aims to provide the same brown appearance [51] or crispy taste [28] as traditional heating methods by utilizing microwave-active materials. The majority of studies on metalized packaging structures only focused on the theoretical analysis, examining the impact of metalized packaging structures on microwave reheating uniformity [11,29,33]. Metalized packages have been typically created using aluminum foil attached on the packaging box, which is unsuitable for industrial production [33,36]. Therefore, an aluminum film solution over a rectangular packaging box to form a metalized packaging was proposed, which is suitable for industrial production [4].

### 3.1. The Analysis of Temperature Distribution in RER Based on the Electric Field Distribution

**Figure 10:**
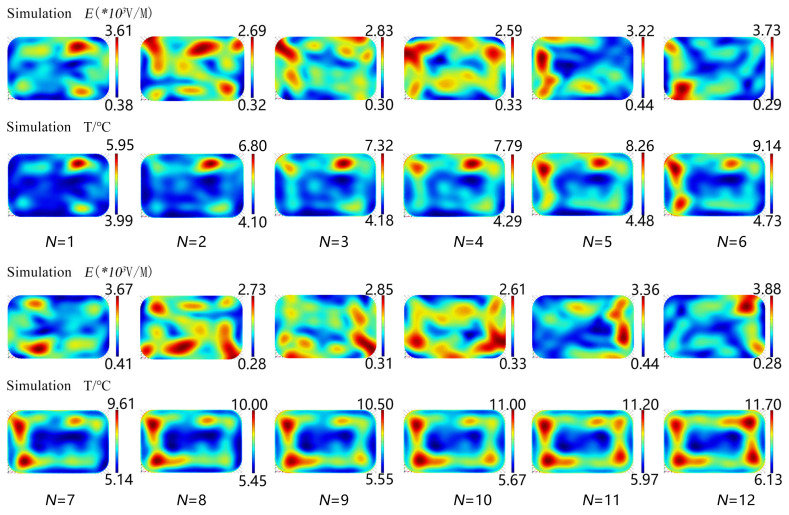
Distribution of the RER’s electric field and temperature at 12 discrete locations during the first rotation cycle. The PP rectangular packaging box had a bottom size of 138 × 86 mm^2^ and a height of 44 mm. The microwave power was set at 800 W and the microwave frequency was set at 2.45 GHz.

The electric field and temperature distribution of 12 sampling sites in layer B_1_ were studied through the simulation model’s first cycle to investigate influence on the temperature distribution inside the RER under microwave reheating (Figure 10). In the first discrete position, the temperature distribution followed the trend of the electric field distribution, with a high temperature appearing in the same position as a high electric field. The electric field distribution in the TE model was parallel to layer B_1_, and it dominated the temperature distribution in layer B_1_. The RER in a rectangular packaging box would undergo an uneven electric field as it rotated, and the microwave volume heat superposition created was similar in the circumferential direction along the rotation path of the packaging box, while the temperature decreased along the rotation radial direction from the packaging box’s border to the center [11]. As a result, the temperature distribution in layer B_1_ gradually diverged from the electric field distribution at the current position, with the fastest rate of temperature rise occurring at the discrete place with the highest electric field. When the glass turntable containing a rectangular packaging box rotated within the microwave oven, the RER temperature progressively rose in the corners and decreased in the center.

During microwave reheating, the changes in the electric field distribution at each sampling position depended on the relative position of the RER and the waveguide [41,52]. Furthermore, due to the microwave reflection of the metal cavity wall, a standing wave was easily formed in the microwave oven, resulting in a multi-mode electric field distribution within the structure of the RER [11]. The multi-mode electric field could be divided into TE waves parallel to the *x*-*y* plane (Figure 6) of the rectangular RER and TM waves perpendicular to it, according to Freanel’s law [11]. The interaction between the TE wave with the edges inside the RER in a rectangular packaging box caused microwave scattering and non-resonant reflection, resulting in a high electric field near the edge of the RER. As a dielectric material, the RER caused the microwave cavity’s TM wave to enter vertically from its surface (*x*-*y* plane), generating an electric current according to Faraday’s law and producing heat near the packaging box’s edge. The TE wave entered the RER parallel to its surface (*x*-*y* plane) and was distributed differentially across its surface as sine or cosine (depending on the wave’s phase) [4,11]. Due to the attenuation of the TE wavelength inside the RER, the electric field distribution generates the alternating strength with the interval at half of a wavelength [11]. As a result, the temperatures inside the RER exhibit ‘cold spots’ and ‘hot spots’ [50]. And as the microwave penetrates the RER, it could create significant attenuation. Because the vertical distance between the bottom of the packaging box and the bottom of the microwave cavity was less than half a wavelength, the packaging box’s metal wall reflected the electromagnetic wave, resulting in a longitudinal magnetic field. The longitudinal magnetic field generated heat by interacting with the RER, which caused heating at the bottom of the packaging box. As a result, the rectangular packaging box had a high electric field intensity distribution along its sides and corners, and the bottom of the packaging box was heated. The RER’s temperature distribution was identical to the electric field distribution during the early stages of heating. Because the RER rotated at a constant rate in the microwave oven, it was difficult to predict the heating temperature trend using a single distribution of the electric field. The electric field of distinct modes at each position had the greatest impact on the temperature distribution under microwave reheating. As a result, the RER’s ‘wall and corner effects’ were produced via a combination of electric field rotation and superposition.

### 3.2. The Influence of Metalized Packaging on RER Reheating

#### 3.2.1. Identifying RER’s Metalized Packaging Structure

**Figure 11:**
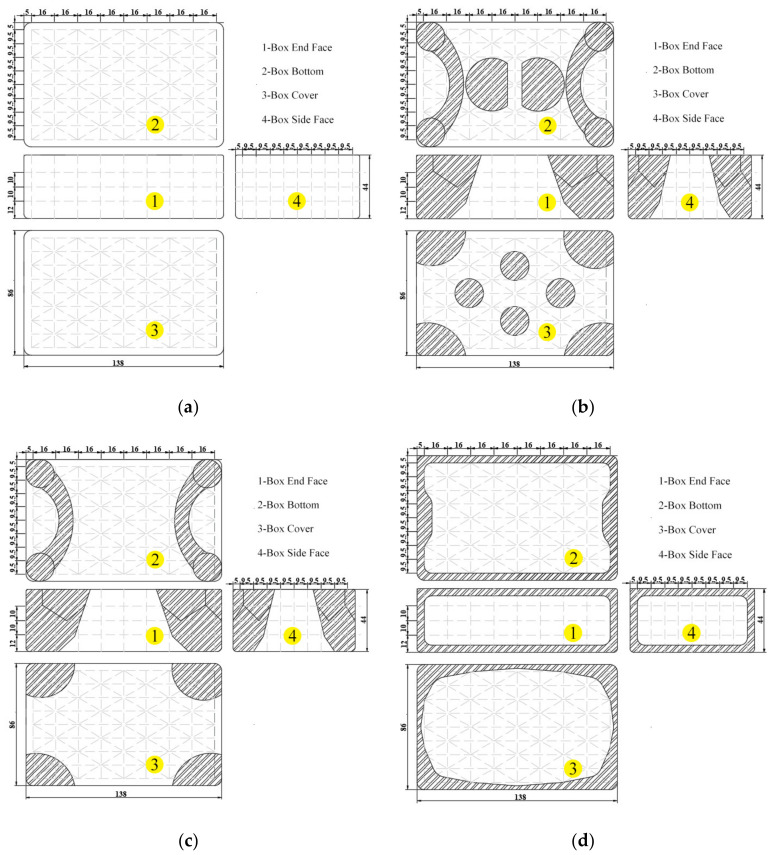
The structure design of the metalized packaging box with an aluminum film thickness of 0.30 mm [4]. (**a**) The metalized packaging structure design of model *I*; the structure was designed without aluminum films as a non-metalized packaging. (**b**) The metalized packaging structure design of model *II*; the structure was designed based on the temperature distribution of RER during microwave reheating with a power of 800 W, and the reheating time was 180 s [4]. (**c**) The metalized packaging structure design of model *III*; the structure removed the center aluminum films from the box cover and bottom found in model *II*. (**d**) The metalized packaging structure design of model *IV*; the structure reduced the width of the metalized pattern at the edge and corner of the packaging box compared to model *III* and connected all aluminum films.

According to the bench test of microwave-reheated RER, the microwave reflection in the microwave oven cavity caused the standing wave effect and insufficient microwave penetration depth with a distinct uneven heating phenomenon [4]. A metal packaging structure for RER was designed to enhance the temperature distribution uniformity. In previous research, we designed a metalized packaging structure to ameliorate the temperature non-uniformity [4]. The metalized packaging was developed to take advantage of the microwave-active material’s microwave reflection properties (aluminum film) to properly adjust the electric field distribution within the RER [4,29,42]. The metalized packaging structure of model *I* was designed based on the temperature distribution of the RER during microwave reheating in the *x*-, *y*-, and *z*-directions, where *x* and *y* were the long and short sides of the packaging box, respectively, and *z* was the direction of the TE transverse wave propagation [4,44]. The aluminum film’s thickness was 0.30 mm [4]. The metalized packaging structures for the RER simulation models were designed as illustrated in Figure 11.

(1) The packaging box of model *I* was not metalized, as shown in Figure 11a.

(2) The packaging box of model *II* was designed with the metalized packaging structure, depicted in Figure 11b. Metalization was used on the corners, the cover center, and the bottom center of the packaging box. Due to a focus on the uniformity of the temperature distribution inside the reheated RER, obvious wall and corner effects occurred. The metalized packaging structure was intended to induce the microwave to reflect at the ‘hot spots’, reducing the ‘hot spots’ temperature while increasing the ‘cold spots’ temperature, meeting the goal of enhancing the temperature distribution uniformity [4].

(3) The packaging box of model *III* eliminated the metalized designs at the center of the box cover and box bottom found in model *II*, as illustrated in Figure 11c. The metalized packaging structure of model *II* was covered by large pieces of aluminum film, and the ‘hot spots’ of the corners were easily converted into ‘cold spots’, which could not meet the purpose of enhancing the uniformity of reheating, and the packaging box of model *III* was designed to avoid this issue.

(4) The packaging box of model *IV* reduced the width (set to 5 mm) of the metalized pattern at the edge and corner of the packaging box compared to model III, as shown in Figure 11d.

**Figure 12:**
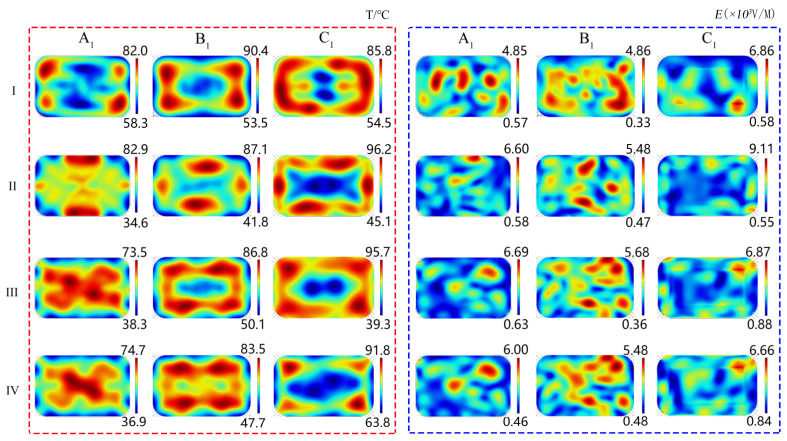
The temperature and electric field distribution of the RER’s A_1_, B_1_, and C_1_ layers in four metalized packaging model types (model *I*, model *II*, model *III*, and model *IV*) after 180 s of microwave reheating. The PP rectangular packaging box had a bottom size of 138 × 86 mm^2^ and a height of 44 mm. The microwave power was 800 W, the reheating time was 180 s, and the microwave frequency was 2.45 GHz. The roman numerals in the figure represented for four metalized packaging model types respectively (model *I*, model *II*, model *III*, and model *IV*).

**Figure 13:**
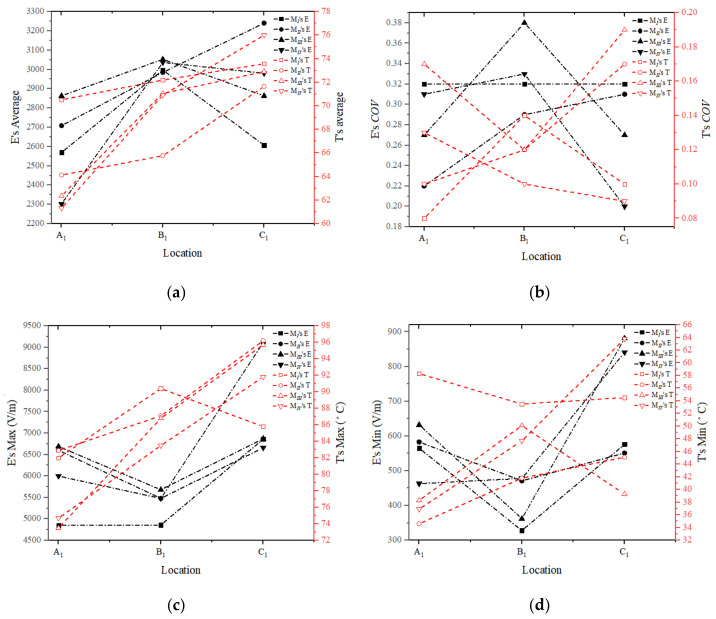
The uniformity of temperature and electric field distribution in the model *I*, *II*, *III*, and *IV*. (**a**) The electric field and temperature average in model *I*, *II*, *III*, and *IV*; (**b**) the uniformity coefficient of the electric field and temperature in model *I*, *II*, *III*, and *IV*; (**c**) the maximum of the electric field and temperature in model *I*, *II*, *III*, and *IV*; (**d**) the minimum of the electric field and temperature in model *I*, *II*, *III*, and *IV*. The temperature or electric field were measured at the RER’s A_1_, B_1_, and C_1_ layers according to the arrangement scheme of measurement points (Figure 6) after reheating with a microwave power of 800 W and a reheating time of 180 s (Figure 12).

Figure 12 depicts the temperature and electric field distribution of the RER’s A_1_, C_1_, and B_1_ layers for a 180 s microwave reheating model, while Figure 13 shows the uniformity of the temperature and electric field distribution. The change in the electric field distribution inside the RER under the microwave reheating process resulted from the usage of a metalized packaging design with a decrease in the maximum electric field and an increase in the minimum electric field. Furthermore, the utilization of a metalized packaging design altered the distribution of ‘hot spots’ and ‘cold spots’ in the RER’s temperature field, enhancing the temperature distribution uniformity, which is consistent with previous experimental findings [4].

The maximum temperature and minimum temperature of the RER decreased. However, due to the usage of a metalized packaging design in models *II*, *III*, and *IV*, the maximum temperature transferred from the ‘hot spots’ of model I to its ‘cold spots’ area, whereas the minimum temperature appeared in the corners of the packaging box (Figure 12). The average temperature and uniformity of models *II* and *III* declined. The difference between model *IV*’s maximum and minimum values decreased, while the temperature distribution uniformity was enhanced.

Since the electric field was only instantaneous, the research on the electric field distribution uniformity focused solely on evaluating whether employing metalized packaging enhances this property, and if so, how this improvement in uniformity affected the temperature distribution uniformity. The metalized packaging structure of models *II*, *III*, and *IV* could improve the uniformity of the electric field distribution. The temperature distribution resulted from the accumulation of heat energy during the 180 s microwave reheating procedure. The temperature distribution enables a more precise comparison among the four simulation models. In comparison to the average temperature, temperature distribution uniformity, and temperature extremes of four models, Model *IV* had a superior temperature distribution that was closer to the desired outcome.

**Figure 14:**
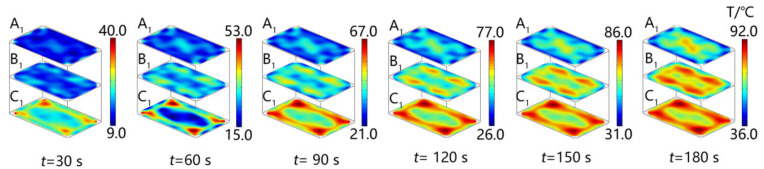
The temperature distribution in the A_1_, B_1_, and C_1_ layers (*x*-*y* plane) of model *IV*. The RER had a moisture content of 63.23 ± 0.87% [4]. The PP rectangular packaging box had a bottom size of 138 × 86 mm^2^ and a height of 44 mm. The microwave power was 800 W, the microwave frequency was 2.45 GHz, and the reheating times were 30, 60, 90, 120, 150, and 180 s, respectively.

The metalized packaging structure of model *IV* improved the temperature distribution uniformity throughout the RER’s microwave reheating process. To enhance the temperature distribution uniformity and energy efficiency inside the RER through model *IV*, it was necessary to obtain the temperature distribution during microwave reheating. Figure 14 depicts the temperature distribution of the RER’s A_1_, B_1_, and C_1_ layers along the *z* direction (*x*-*y* plane) for reheating times of 30, 60, 90, 120, 150, and 180 s. The aluminum film in model *IV* could be used to remove ‘hot spots’ in the corners of packaging boxes, diminish the region of ‘cold spots’, and lessen temperature differentials between ‘hot spots’ and ‘cold spots’. The model *IV* enhanced the uniformity of the temperature distribution during the microwave reheating process.

#### 3.2.2. The Microwave Reheating Effect Verification of Metalized Packaging

A microwave reheating experiment was conducted to verify the control effect of the metalized packaging on the RER temperature distribution uniformity, and a comparison was made between the impacts of the RER’s temperature distribution in the model *I* and model *IV* metalized packaging. The RER’s metalized packaging was constructed using an aluminum film solution, and the aluminum film pattern was sprayed at 3.5 × 10^−4^ mL/mm^2^, with an aluminum film thickness of 0.03 mm [4].

**Figure 15:**
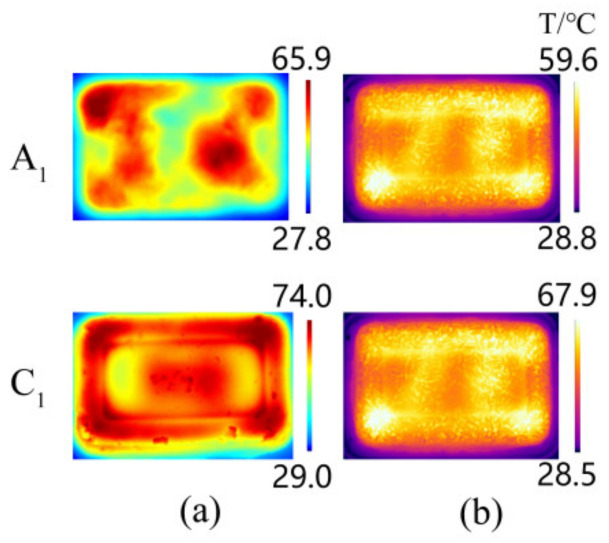
Temperature distribution on the A_1_ and C_1_ layers of RER after microwave reheating in a verification experiment for model *I* and model *IV*. (**a**) The temperature distribution of A_1_ and C_1_ layers in a microwave reheating verification experiment of model *I*; (**b**) the temperature distribution of A_1_ and C_1_ layers in a microwave reheating verification experiment of model *IV*. The RER had a moisture content of 63.23 ± 0.87% [4]. The PP rectangular packaging box had a bottom size of 138 × 86 mm^2^ and a height of 44 mm. The microwave power was 800 W, the reheating time was 180 s, and the microwave frequency was 2.45 GHz.

**Figure 16:**
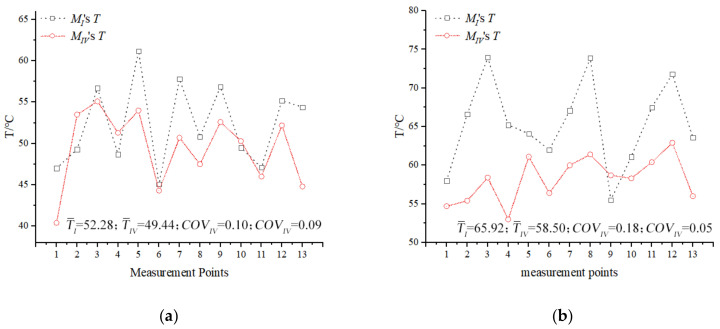
Temperature distribution of 13 points (Figure 6) in the A_1_ and C_1_ layers after microwave reheating in a microwave reheating verification experiment for model *I* and model *IV*. (**a**) The temperature distribution of 13 points in the A_1_ layer after microwave reheating in a microwave reheating verification experiment for model *I* and model *IV*; (**b**) the temperature distribution of 13 points in the C_1_ layer after microwave reheating in a microwave reheating verification experiment for model *I* and model *IV*.

Figure 15 depicts the temperature distribution of the A_1_ and C_1_ layers in the corresponding bench tests of model *I* and model *IV* after 180 s of reheating. In comparison to model *I*, the experimental temperature of model *IV*’s A_1_ and C_1_ layers increased by 8.03 and 7.42 °C, respectively. The temperature uniformity increased by 35.71% and 72.22%, respectively. Using metalized packaging could significantly improve the uniformity of the temperature distribution during the microwave reheating of RER (Figure 16).

## 4. Conclusions

The effects of the metalized packaging structure on the temperature uniformity in RER under microwave reheating were analyzed through dynamic simulation and bench experiments. The main findings are as follows.

(1) The proposed metal packaging structure enhances the temperature distribution uniformity in the RER under microwave reheating based on an optimal metal aluminum film, with a width of 5 mm and a thickness of 0.30 mm in the corner of the container. The design improves the temperature uniformity in the RER, and this is attributed to the even distribution of the electric field in the metal packaging structure.

(2) For the RER loaded in a metal packaging container, the microwave-generated heat around the edges is attenuated and focused in the case of rectangular-shaped RER packages, which influences the uniformity of the interior temperature due to the reflection of the aluminum film in the microwave.

The research results were applied to ready-to-eat meals in rectangular packaging boxes and could provide a theoretical basis for the design of microwave-heated food packaging boxes for multi-dish ready-to-eat meals. During circulation, the safety of food in metalized packaged ready-to-eat meals should also be investigated further.

## Figures and Tables

**Table 1:** Physical properties of air, glass turntable, copper, and aluminum.

Attribute	Materials	Value
Relative dielectric constant	Air	1
	Glass turntable	4.2
	Copper	1
	Aluminum	1
Relative permeability	Air	1
	Glass turntable	1
	Copper	1
	Aluminum	1
Conductivity (s/m)	Air	0
	Glass turntable	1 × 10^−14^
	Copper	5.998 × 10^7^
	Aluminum	2.326 × 10^7^

**Table 2:** The physical properties list of RER [4]. The value formulas of thermal conductivity, specific heat, dielectric constant, dielectric loss, and penetration depth for RER were fitted for the temperature range of 10–80 °C.

Material Parameter	Value	Unit
Apparent density	880	$\mathrm{kg}/m^{3}$
Absolute density	1670	$\mathrm{kg}/m^{3}$
Porosity	47.31	%
Water content	63.23	%
Thermal conductivity	$K=0.5080-0.0066\times +1.4152\times 10^{-4}\times T^{2}$	W/mk
Specific heat	$C=3.3069+0.0037T+1.4596\times 10^{-5}T^{2}-5.3294\times 10^{-7}T^{3}$	$\mathrm{MJ}/m^{3}k$
Dielectric constant	$\varepsilon^{\prime }=47.7486-0.6258T+0.0930T^{2}-0.0034T^{3}\;+4.7759\times 10^{-5}T^{4}-2.3331\times 10^{-7}T^{5}$	1
Dielectric loss	$\varepsilon^{\prime \prime }=11.8675+0.0405T-0.0038T^{2}+3.4296\times 10^{-5}T^{3}$	1
Penetration depth	$d=11.2973-0.0284T+0.0049T^{2}-4.8771\times 10^{-5}T^{3}$	mm

**Table 3:** Data values for mesh generation and sizing.

Mesh Number in a Single Wavelength	Maximum Size of Different Material Units				Mesh Number	Time (s)
	RER	Turntable	Cavity	Dielectric		
0.5	53.78	119.42	244.73	173.04	80,659	995
1	26.89	59.71	122.37	86.52	82,120	976
2	13.45	29.86	61.19	43.26	84,843	849
3	8.97	19.91	40.79	28.84	98,475	601
4	6.73	14.93	30.6	21.63	135,313	710
5	5.38	11.95	24.48	17.30	195,884	950
6	4.49	9.96	20.4	14.42	290,244	1376
7	3.85	8.53	17.49	12.36	428,532	1910
8	3.37	7.47	15.3	10.81	593,810	3206
9	2.99	6.64	13.6	9.61	1,084,421	4286
10	2.69	5.98	12.24	8.65	1,448,047	5440
11	2.45	5.43	11.13	7.86	1,454,705	9656

## Data Availability

The original contributions presented in the study are included in the article, further inquiries can be directed to the corresponding author.

## References

[1] Montero M.L., Garrido D., Gallardo R.K., Tang J., Ross C.F. (2021). Consumer Acceptance of a Ready-to-Eat Meal during Storage as Evaluated with a Home-Use Test. Foods.

[2] Fan D.C., Xu J.C., Zhang M., Chen J.J. (2022). Improved Flavor of Instant Rice for Airline Catering. J. Food Sci. Biotechnol..

[3] Chandrasekaran S., Ramanathan S., Basak T. (2013). Microwave Food Processing—A Review. Food Res. Int..

[4] Liu C., Shen L., Liu H., Gong X., Liu C., Zheng X., Zhang S., Yang C. (2023). Improvement of Temperature Distribution Uniformity of Ready-to-Eat Rice during Microwave Reheating via Optimizing Packaging Structure. Foods.

[5] Zhang Z., Su T., Zhang S. (2018). Shape Effect on the Temperature Field during Microwave Heating Process. J. Food Qual..

[6] Fan D.M., Chen W., Li C.X., Wang L.Y., Pang K., Zhao J.X., Zhang H. (2012). Size effect on temperature distribution of instant rice during microwave reheating process. Trans. Chin. Soc. Agric. Eng..

[7] Cao H., Fan D., Jiao X., Huang J., Zhao J., Yan B., Zhou W., Zhang W., Ye W., Zhang H. (2019). Importance of Thickness in Electromagnetic Properties and Gel Characteristics of Surimi during Microwave Heating. J. Food Eng..

[8] Cao H., Fan D., Jiao X., Huang J., Zhao J., Yan B., Zhou W., Zhang W., Zhang H. (2018). Heating Surimi Products Using Microwave Combined with Steam Methods: Study on Energy Saving and Quality. Innov. Food Sci. Emerg. Technol..

[9] Zheng X.Z., Lu T.L., Chen Q.M., Zhang Y.H., Shen L.Y., Fu K.S., Zhu H.H., Bai C.Y. (2024). Effects of compound additives on the physicochemical properties of low-temperature spray drying Loniceraedulis powder. Trans. Chin. Soc. Agric. Eng..

[10] Wang L., Zhao Y., Ma W., Shen L., Liu C., Liu C., Zheng X., Li S. (2022). Utilization Efficiency of Microwave Energy for Granular Food in Continuous Drying: From Propagation Properties to Technology Parameters. Dry. Technol..

[11] Ulrich E. (2020). Development of Packaging and Products for Use in Microwave Ovens.

[12] Suvi R. (2002). Microwave Heating Uniformity of Multicomponent Prepared Foods. Ph.D. Thesis.

[13] Zhu W.X., Nie Z.H., Wang H.D., Wang L. (2022). Simulation on Temperature Field of Microwave Reheating of Cold-chain Quick-frozen Food with Different Packaging Materials. Packag. Eng..

[14] Wang X.Q., Du P., Wang X.Y. (2014). Temperature Distribution on Cylindrical Shaped Food during Microwave Heating. Sci. Technol. Eng..

[15] Dai M.H., Guo W., Cheng Y.D., Jin Y.Z. (2015). Three—Dimensional temperature distribution of the packaged foods with different shapes during microwave heating. Sci. Technol. Food Ind..

[16] Pitchai K., Chen J., Birla S., Gonzalez R., Jones D., Subbiah J. (2014). A Microwave Heat Transfer Model for a Rotating Multi-Component Meal in a Domestic Oven: Development and Validation. J. Food Eng..

[17] Miran W., Palazoğlu T.K. (2019). Development and Experimental Validation of a Multiphysics Model for 915 MHz Microwave Tempering of Frozen Food Rotating on a Turntable. Biosyst. Eng..

[18] Huang Z., Datta A.K., Wang S. (2018). Modeling Radio Frequency Heating of Granular Foods: Individual Particle vs. Effective Property Approach. J. Food Eng..

[19] Klinbun W., Rattanadecho P. (2023). A Computational Analysis of How the Design of Multicompartment Containers and Placement Angle Affect Heat and Mass Transfer during the Microwave Heating Process. Eng. Sci..

[20] Zhu H., He J., Hong T., Yang Q., Wu Y., Yang Y., Huang K. (2018). A Rotary Radiation Structure for Microwave Heating Uniformity Improvement. Appl. Therm. Eng..

[21] Yang R., Morgan M., Fathy A., Luckett C., Wang Z., Chen J. (2023). A Comprehensive Evaluation of Microwave Reheating Performance Using Dynamic Complementary-Frequency Shifting Strategy in a Solid-State System. Food Bioprocess. Technol..

[22] Fia A.Z., Amorim J. (2021). Heating of Biomass in Microwave Household Oven—A Numerical Study. Energy.

[23] Du Z., Wu Z., Gan W., Liu G., Zhang X., Liu J., Zeng B. (2019). Multi-Physics Modeling and Process Simulation for a Frequency-Shifted Solid-State Source Microwave Oven. IEEE Access.

[24] Zhang S., Ramaswamy H., Wang S. (2019). Computer Simulation Modelling, Evaluation and Optimisation of Radio Frequency (RF) Heating Uniformity for Peanut Pasteurisation Process. Biosyst. Eng..

[25] Jeong C.H., Ahn S.H., Lee W.S. (2019). Four-kilowatt Homogeneous Microwave Heating System Using a Power-controlled Phase-shifting Mode for Improved Heating Uniformity. Electron. Lett..

[26] Zhou X., Pedrow P.D., Tang Z., Bohnet S., Sablani S.S., Tang J. (2023). Heating Performance of Microwave Ovens Powered by Magnetron and Solid-State Generators. Innov. Food Sci. Emerg. Technol..

[27] Wang G., Meng J., Zhang K. (2024). Process Intensification of Non-Uniform Additive Pattern for Coal Slime Drying by Microwave Heating. Energy.

[28] Chen F., Warning A.D., Datta A.K., Chen X. (2017). Susceptors in Microwave Cavity Heating: Modeling and Experimentation with a Frozen Pie. J. Food Eng..

[29] Song W.H., Wang R.F., Li Z.Y., Xu Q. (2014). Improvement of electrically conductive bead on partial overheating phenomenon in microwave food heating. Food Mach..

[30] Ghimire A., Yang R., Chen J. (2024). The Combined Effect of Active Packaging and Relative Phase Sweeping on Microwave Heating Performance in a Dual-Port Solid-State System. J. Microw. Power Electromagn. Energy.

[31] Prosetya H., Datta A. (2017). Batch Microwave Heating of Liquids: An Experimental Study. J. Microw. Power Electromagn. Energy.

[32] Dou R.B. (2013). Effect of Electrically Condrctive Beads on the Electromagnetic Field Distribution in a Microwave Cavity. Master’s Thesis.

[33] Zhang K. (2014). Metallized Microwave Packaging Research to Improve the Effect of Microwave Heating of Food. Master’s Thesis.

[34] Lai L.M.C., Zeng N., Liu B. (2013). Uniformly Heated Microwave Container. China Patent.

[35] Ho Y.C., Yam K.L. (1992). Effect of Metal Shielding on Microwave Heating Uniformity of a Cylindrical Food Model. J. Food Process. Preserv..

[36] Wang X.R. (2018). Study on the Temperature Distribution of Instant Rice During Microwave Reheating. Master’s Thesis.

[37] Chen J.M. (2014). Research on Quality Control of Packed Rice During Hot Chain and Reheating of Cold Chain. Master’s Thesis.

[38] Chen D.W., Zhang Y., Luo M.L. (2018). The effect of home microwave oven on the nutritional components of cooked rice. Grain Processing.

[39] Song Y.Y. (2011). Studies on the Quality Holding and Shelf Life of Colde Distributional Cooked Rice. Master’s Thesis.

[40] Shen L.Y., Zhu Y., Liu C.H., Wang L., Liu H., Kamruzzaman M., Liu C., Zhang Y.P., Zheng X.Z. (2020). Modelling of Moving Drying Process and Analysis of Drying Characteristics for Germinated Brown Rice under Continuous Microwave Drying. Biosyst. Eng..

[41] Tepnatim W., Daud W., Kamonpatana P. (2021). Simulation of Thermal and Electric Field Distribution in Packaged Sausages Heated in a Stationary Versus a Rotating Microwave Oven. Foods.

[42] Liu Z.M., Du H., Shi N.L., Wen L.S. (2008). Influence of Conductivity Size Effect on the Microwave Absorption Properties of Aluminium Films. Acta Metall. Sin..

[43] Bhattacharya M., Basak T. (2016). A Review on the Susceptor Assisted Microwave Processing of Materials. Energy.

[44] Zhu Y. (2021). Study on Improving Temperature Uniformity of Microwave-Reheated Instant Rice with Metallized Packaging Film. Master’s Thesis.

[45] Pitchai K., Birla S.L., Subbiah J., Jones D., Thippareddi H. (2012). Coupled Electromagnetic and Heat Transfer Model for Microwave Heating in Domestic Ovens. J. Food Eng..

[46] Ye J.H., Zhu H.C., Liao Y.H., Zhou Y.P., Huang K.M. (2017). Implicit Function and Level Set Methods for Computation of Moving Elements During Microwave Heating. IEEE Trans. Microw. Theory Tech..

[47] Chen J., Pitchai K., Jones D., Subbiah J. (2015). Effect of Decoupling Electromagnetics from Heat Transfer Analysis on Prediction Accuracy and Computation Time in Modeling Microwave Heating of Frozen and Fresh Mashed Potato. J. Food Eng..

[48] Sun Y., Sha W.X., Liu W., Huang N.J., Zhang B.N., Zhou X.Y. (2024). Effect of Stewing Time on Edible Quality of Rice. Food Res. Dev..

[49] Geedipalli S.S.R., Rakesh V., Datta A.K. (2007). Modeling the Heating Uniformity Contributed by a Rotating Turntable in Microwave Ovens. J. Food Eng..

[50] Wang L., Shen L.Y., Liu C.H., Liu C., Zheng X.Z. (2021). Effect of electric field distribution on energy use efficiency for berry puree under microwave drying. Trans. Chin. Soc. Agric. Eng..

[51] Gu M.M., Yang N., Xu X.M. (2012). Study on the properties of breads baked by microwave with susceptor. Sci. Technol. Food Ind..

[52] Shen L.Y., Gao M., Feng S.X., Ma W.Y., Zhang Y.H., Liu C.H., Liu C., Zheng X.Z. (2022). Analysis of Heating Uniformity Considering Microwave Transmission in Stacked Bulk of Granular Materials on a Turntable in Microwave Ovens. J. Food Eng..

